# Centipede Venoms and Their Components: Resources for Potential Therapeutic Applications

**DOI:** 10.3390/toxins7114832

**Published:** 2015-11-17

**Authors:** Md Abdul Hakim, Shilong Yang, Ren Lai

**Affiliations:** 1 Key Laboratory of Animal Models and Human Disease Mechanisms, Kunming Institute of Zoology, Chinese Academy of sciences, Kunming 650223, Yunnan, China; E-Mails: hakeem.geb.ru@gmail.com (M.A.H.); yslzoology@163.com (S.Y.); 2 University of Chinese Academy of Sciences, Beijing100009, China; 3 Joint Laboratory of Natural Peptide, University of Science and Technology of China and Kunming Institute of Zoology, Chinese Academy of Sciences, Kunming 650223, China

**Keywords:** centipedes, venom, peptides, toxins, pharmacology, therapeutics

## Abstract

Venomous animals have evolved with sophisticated bio-chemical strategies to arrest prey and defend themselves from natural predators. In recent years, peptide toxins from venomous animals have drawn considerable attention from researchers due to their surprising chemical, biochemical, and pharmacological diversity. Similar to other venomous animals, centipedes are one of the crucial venomous arthropods that have been used in traditional medicine for hundreds of years in China. Despite signifying pharmacological importance, very little is known about the active components of centipede venoms. More than 500 peptide sequences have been reported in centipede venomous glands by transcriptome analysis, but only a small number of peptide toxins from centipede has been functionally described. Like other venomous animals such as snakes, scorpions, and spiders, the venom of centipedes could be an excellent source of peptides for developing drugs for treatments as well as bio-insecticides for agrochemical applications. Although centipede venoms are yet to be adequately studied, the venom of centipedes as well as their components described to date, should be compiled to help further research. Therefore, based on previous reports, this review focusses on findings and possible therapeutic applications of centipede venoms as well as their components.

## 1. Introduction

Centipedes, class Chilopoda, are one of the oldest extant terrestrial arthropods. Approximately 3300 species of centipede have been described in five extant orders [1,2]. Among them, a larger numbers and species of centipede are found in tropical and subtropical countries. Centipedes are venomous animals possessing strong mandibles and venomous fangs, called forcipules, which stem from the first pair of legs. The *Scolopendra* species (Scolopendromorpha: Scolopendridae) is the best-known genus of centipede [3].

Behaviorally, centipedes are predatory animals [4,5] that capture mostly vertebrates—including reptiles, amphibians, rats and bats, as well as several types of insects [1,6]—by using their venom as a potent weapon [6,7]. To protect from prey and defend against their predators, centipedes secrete venoms from their venom glands connected to the first pair of forcipules [8]. Venom is secreted specifically via a pore located near the tip of each forcipule [9]. Notably, the venom-injecting forcipules of centipedes show an evolutionary novelty that appeared in the centipede stem lineage more than 400 Mya. No other lineage of arthropods or animals has evolved claws for injecting venom from a pair of walking legs [10].

Based upon symptoms and complications induced by centipede envenomation, it has been suggested that the centipede venom comprises a diverse cocktail of toxins [1]. Similar to other venoms, such as snake or scorpion, the venoms of centipedes are a natural pool of proteins, peptides and enzymes with a rich diversity of biological activities. Recent studies have indicated that venoms from a single centipede contain more than 500 proteins and peptides [11,12,13,14,15]. The active components of centipede venom which can rapidly paralyze prey are mostly neurotoxic proteins and peptides [16]. However, little is known about the venom components as well as their mechanism of action. A study on *Scolopendra subspinipes mutilans* L. Koch venom peptides has recently been methodically performed. It revealed that the neurotoxins present in the venom of this centipede acts on ion channels to cause rapid paralysis [12]. In addition, transcriptomic and proteomic analysis along with biological functional assays on *S. subspinipes dehaani* venom has demonstrated that the main fractions of crude venom contain neurotoxic components that act on ion channels [11]. Thus far, around 50 components of centipede venom have been reported with the following pharmacological properties: voltage-gated sodium channel activities, voltage-gated potassium channel activities, voltage-gated calcium channel activities, TRPV1 channel activity, platelet-aggregating activity, anticoagulant activity, phospholipase A2 activity, and trypsin-inhibiting activity [11,12,13].

Centipedes have been used for hundreds of years in traditional Chinese medicine to treat many disorders, such as stroke-induced hemiplegia, epilepsy, apoplexy, whooping cough, tetanus, burns, tuberculosis, as well as myocutaneous disease. Furthermore, centipedes have been described for the treatment of cardiovascular diseases for several hundred years in Korea, China, and other Far Eastern Asian countries [17,18]. These historical and ethnopharmacological references indicate that centipede venom and its components could be exploited for therapeutic use and drug development.

According to the previous reviews and published reports, centipedes are venomous arthropod lineages which are very different from snakes or scorpions. The venoms of snakes or scorpions have already been extensively studied; however, venoms of centipedes remain less explored. In spite of their abundance and frequent encounters with humans (often involving painful bites), very few studies on the components of centipede venom have been carried out, thus signifying that more research is necessary. Importantly, components from centipede venom reported to date could be screened for potential therapeutic uses. To help unveil further therapeutic applications, we describe known centipede venoms and their proteins/peptides with pharmacologically interesting activities. These include ion channel modulators, antimicrobial peptides, different enzymes, enzyme inhibitors, anticancer peptides, antithrombotic peptides, as well as anticoagulants and centipede extracts.

## 2. Pharmacologically Important Proteins/Peptides from Centipedes

The venom components of centipedes remained almost completely untouched for investigational purposes [1,19]. During the past recent years, some proteins/peptides have been identified from centipedes and a few of them have been described with important pharmacological properties, which are compiled and categorized here as follows.

### 2.1. Ion Channel Modulators

Toxins from venomous animals such as snakes, scorpions and spiders have been well-investigated for ion channels. A large number of toxins from venomous animals have been reported to target different types and subtypes of ion channels [20,21,22,23,24,25,26,27,28]. However, centipede venom and its components have not yet been extensively characterized. Here we describe the ion channel-modulating toxins identified to date from centipede venoms. Yang *et al*. were the first to describe molecules from the Chinese red-headed centipede *S. subspinipes mutilans*, which act as modulators of voltage-gated sodium (Na_V_), potassium (K_V_), and calcium (Ca_V_) channels. They provided the first conclusive evidence that centipede venoms were replete with bioactive cysteine-rich peptides. First, a neurotoxin peptide with a molecular weight of 3762.5 Da, named µ-SLPTX-Ssm1a, was found to specifically inhibit tetrodotoxin-sensitive (TTX-S) Na_V_ channels with an IC_50_ of ~9 nM. In their work, three other peptide neurotoxins were purified and found to inhibit K_V_ channel currents in dorsal root ganglion (DRG) neurons. First, neurotoxin peptide with a molecular weight of 6050.2 Da, named κ-SLPTX-Ssm1a, was found to inhibit the K_V_ channel currents with an IC_50_ of 44.2 ± 5.7 nM. The second K_V_ channel modulator was named κ-SLPTX-Ssm2a, with a molecular weight of 3465.8 Da, which was found to inhibit K_V_ channel in DRG neurons with an IC_50_ of 570 ± 126 nM. The third neurotoxin, κ-SLPTX-Ssm3a with a molecular weight of 7989.05 Da, was functionally described to inhibit K_V_ channel [12]. It was reported that 200 nM κ-SLPTX-Ssm3a inhibited K_V_ current amplitude in DRG neurons by only 25% ± 5% and, even at concentrations of up to 5 µM, κ-SLPTX-Ssm3a did not inhibit all K_V_ currents. However, this toxin was found to display an obvious difference in its ability to inhibit peak currents and slowly activated delayed rectifier K_V_ currents [12]. Furthermore, two peptide neurotoxins, ω-SLPTX-Ssm1a and ω-SLPTX-Ssm2a, acting on a voltage-gated calcium (Ca_V_) channel were reported by Yang *et al*. ω-SLPTX-Ssm1a, with the molecular weight of 8818.8 Da, was reported to act as an activator of Ca_V_ channel in DRG neurons. Functionally, ω-SLPTX-Ssm1a was found to increase Ca_V_ currents by 70%, whereas application of 10 µM toxin increased Ca_V_ currents by ~120%. In contrast, ω-SLPTX-Ssm2a was described as an inhibitor of Ca_V_ channel currents in DRG neurons. Functionally, 500 nM of ω-SLPTX-Ssm2a was found to inhibit Ca_V_ current amplitude by only 45% ± 5%, whereas application of increased concentration (2.5 µM ω-SLPTX-Ssm2a) inhibited 80% of the Ca_V_ current. The concentration-response has described that ω-SLPTX-Ssm2a inhibits Ca_V_ channel currents in DRG neurons with an IC_50_ value of about 1590 nM.

Most importantly, Yang *et al.* further discovered a selective Na_V_1.7 inhibitor from the Chinese red-headed centipede *S. subspinipes mutilans* venom. They described the identification of a peptide toxin with a molecular weight of 5318.4 Da, μ-SLPTX-Ssm6a, with 46 residues from centipede venom. Based on electrophysiology, μ-SLPTX-Ssm6a was found to potently inhibit Na_V_1.7 with an IC_50_ of ~25 nM. Interestingly, μ-SLPTX-Ssm6a showed more than 150-fold selectivity for Na_V_1.7 over all other human Na_V_ subtypes. Moreover, it has been proven that μ-SLPTX-Ssm6a is a more potent analgesic than morphine in a rodent model of chemical-induced pain, and it is equipotent with morphine in rodent models of thermal and acid-induced pain [9].

Liu *et al.* isolated and characterized nine different fractions from the venom of centipede *S. subspinipes dehaani* by venomic analysis; these fractions may act as voltage-gated potassium channel inhibitors, calcium channel inhibitors and voltage-gated sodium channel inhibitors. Moreover, by bioinformatic analysis, they reported several putative ion channel modulators from the venom gland transcriptome, indicating that K^+^ channel inhibitors were the most divergent groups. Importantly, the most potent K_V_ inhibitor described by them is κ-SLPTX15-Ssd1a (SSD559; KC144556), which irreversibly inhibits K^+^ currents in DRG neurons with an IC_50_ value of 10 nM [11].

Recently, Chen *et al.* purified a novel neurotoxin peptide named SsmTx-I from *S. subspinipes mutilans*, which has been described as a modulator of voltage-gated potassium channel subtype 2.1 (K_V_2.1). SsmTx-I, a toxin of 36 amino acid residues with the molecular weight of 4114.068 Da was reported as a blocker of K_V_2.1 channel. In electrophysiology, SsmTx-I was found to significantly block voltage-gated K^+^ channels in DRG neurons with an IC_50_ value of 200 nM. SsmTx-I was further tested on nine K^+^ channel subtypes expressed in human embryonic kidney 293 (HEK 293) cells, and experimentally SsmTx-I was exhibited to selectively block the K_V_2.1 current with an IC_50_ value of 41.7 nM [6].

Most recently, a peptide named RhTx has been isolated and characterized from the venom of *S. subspinipes mutilans*. RhTx, composed of 27 amino-acid residues, forms a compact polarized molecule with very rapid-binding kinetics and a high affinity for TRPV1. It has been shown that RhTx causes powerful activation by targeting the channel’s heat-activation machinery at normal body temperature. Furthermore, it has been clearly shown that the RhTx–TRPV1 interaction is facilitated by the toxin’s highly charged C terminus, which associates tightly with the charge-rich outer pore region of the channel where it can directly interact with the pore helix and turret [29].

### 2.2. Antimicrobial Peptides

Antimicrobial peptides (AMPs) are short, ribosomally synthesized peptides, which are naturally found in a variety of invertebrate, plant, and animal species [30,31]. Importantly, antimicrobial peptides have been reported to play an important role in innate immune systems of both invertebrates and vertebrates. They are less than 100 amino acids in length and act as the first line to defend microorganism infection [31,32,33,34,35,36,37]. Until now, a large number of antimicrobial peptides have been reported from animals, plants and microorganisms. Amphibians and insects are considered excellent resources to find antimicrobial peptides [38,39,40,41,42,43,44]. Arthropods, especially centipedes, could also be a source for AMPs, since centipedes have innate immunity and they are believed to produce antimicrobial peptides [45,46]. Peng *et al.* were the first to identify and characterize two antimicrobial peptides, scolopin 1 and scolopin 2, from the venom of centipede *S. subspinipes mutilans*. Molecular masses were determined as 2593.9 and 3017.6 Da for scolopin 1 and 2, respectively. Both peptides showed strong antimicrobial activities against microorganisms, including gram-positive and gram-negative bacteria and fungi. In addition, these peptides were found to show moderate hemolytic activity against both human and rabbit red blood cells [47]. Scolopin 1 was further expressed and recombinant peptide showed similar antimicrobial activity [48]. Next, a novel lactoferricin B-like peptide (LBLP) was derived from the whole bodies of adult centipedes, *S. subspinipes mutilans*, and tested for antifungal activity. LBLP with the molecular weight of 2757.6 Da, was found to exert an antifungal and fungicidal activity against *Candida albicans* without hemolysis. Choi *et al*. confirmed the membrane-active mechanism of LBLP showing that the antifungal effect of LBLP on membrane was due to the formation of pores with radii between 0.74 nm and 1.4 nm. It was suggested that LBLP exhibits a potent antifungal activity by pore formation in the membrane, eventually leading to fungal cell death [49]. Recently, a novel antimicrobial peptide, named scolopendin 1, was identified from adult centipedes, *S. subspinipes mutilans*, by RNA sequencing [45]. Scolopendin 1, with a molecular weight of 5269.4 Da, was reported to show antimicrobial activity against the fungus, *C. albicans*, without inducing hemolysis of human erythrocytes. Choi *et al.* performed a reactive oxygen species (ROS) assay, which indicated that scolopendin 1 induced ROS accumulation in *C. albicans* and subsequently confirmed that ROS are a major factor in scolopendin 1-induced fungal cell death [45]. Another antimicrobial peptide, named Scolopendin 2, was reported by Lee *et al.* Scolopendin 2 is a peptide 16 amino acids in length and with a molecular weight of 1780.4 Da, identified from *S. subspinipes mutilans*, which exhibits antimicrobial activities without causing hemolysis of human erythrocytes. Minimal inhibitory concentration assays were carried out against several strains of bacteria and fungi, revealing a potent antimicrobial activity of scolopendin 2. Moreover, the study confirmed that the membrane-active mechanism leads to the formation of pores in the plasma membrane, subsequent leakage of cytoplasmic matrix components and consequent membrane depolarization, ultimately resulting in microbial cell death [50]. Additionally, three other peptides, scolopendrasin I [51], II, and VII, have been mentioned as showing antimicrobial activities [52,53,54]. According to Kwon *et al.* scolopendrasin II was reported to exhibit antibacterial activities against gram-positive and gram-negative bacterial strains, including the yeast *C. albicans* and antibiotic-resistant gram-negative bacteria without hemolytic activity. Moreover, 17 novel peptides from the *S. subspinipes mutilans* transcriptome were biochemically synthesized and evaluated *in vitro* for activity against various bacteria and yeast. Among them, ten synthetic peptides experimentally exhibited broad antimicrobial activity against microbes without causing any toxicity to mouse erythrocytes [31].

### 2.3. Enzymes

#### 2.3.1. Proteases

According to previous reports, many serine proteases have been identified from venomous animals [55,56,57,58,59,60]. A long time ago, some proteases were reported to be present in the venoms of both scolopendrid and cryptopid scolopendromorphs. For example, three different amino acid naphthylamidase-type amino-peptidases were reported from the maxilliped extract of *S. morsitans* [61]. Endo- as well as exo-peptidases, including fibrinolytic but not fibrinogenolytic activity were also found to be present in the venom of the same species [1]. Likewise, precursors encoding trypsin-like serine proteases have been reported as present in the venom gland transcriptome of *S. subspinipes dehaani* [11]. A novel serine protease, namely scolonase, was identified from the tissue of the centipede, *S. subspinipes mutilans* [62]. Purified scolonase, with an apparent molecular weight of 25 kDa, was able to specially hydrolyze arginine over lysine at the cleavage site in several synthetic peptide substrates. Afterwards, the serine protease activity was proposed as present in centipede venom [63]. Recently, two families of serine proteases, named trypsin-like S1 and subtilisin-like S8 proteases, were identified from scolopendrid venoms [15]. S8 proteases were reported for the first time to be present in centipede venom, whereas S1 proteases are widely recruited proteins into animal venoms which can be found in all studied venomous taxa. As venom components, these proteases are thought to be involved in a range of functions, including smooth muscle contraction, vasodilation, anticoagulation and immunosuppression [15,64,65,66,67,68]. Based on the previous reports, S1 transcripts were found in all five tested species of centipedes, including the venom-gland transcriptome of *S. subspinipes dehaani* [11,15]. Likewise, according to the published report, transcripts of subtilisin-like S8 protease were observed in the venom gland transcriptomes of all scolopendrid species, while they were proteomically identified at a lower level in the venoms of *E. rubripes* and *C. westwoodi* [15]. Furthermore, the weak evidence for the presence of serine proteases was observed in the venom of *Otostigmus pradoi*, suggesting that toxins might be activated either during storage in the extracellular space or upon venom ejection [15].

#### 2.3.2. Metalloproteases

Activity as well as sequence-based investigations have revealed that metalloproteases are important components of centipede venoms [15,19,63]. Based on transcriptomic and proteomic analyses of the venom proteome of *Thereuopoda longicornis* (Scutigeromorpha, Scutigeridae), about 10% of proteins identified from venom are thought to be astacin-like metalloendoproteases (MEROPS family M12, subfamily A) [15,19]. A previous study had set out to propose the metalloproteases in scolopendrid venoms as members of the M12A subfamily; however, M12A proteases appeared to be an ancestral characteristic of centipede venoms [19]. Since metalloproteases are reportedly involved in skin damage, blister formation, edema, myonecrosis and inflammation, the presence of metalloproteases in the centipede indicates consistence with some of the persistent symptoms associated with centipede bites [1,15,69].

#### 2.3.3. Esterases

Non-specific esterases, including four α-naphthyl acetate-positive esterases, four alkaline phosphatases and three acid phosphatases, have been identified from the maxilliped extract of *Scolopendra morsitans*, [1,61,70]. These esterases are assumed to play a role in the release of endogenous purines during envenomation, which then act as multitoxins causing a multitude of pharmacological effects including immobilization through hypotension [71,72].

#### 2.3.4. Phospholipase A_2_

Phospholipase A_2_ (PLA_2_) has been reported to be present as recruited extensively into animal venoms. These enzymes show a diverse array of catalytic and derived non-catalytic activities [64,73]. In centipedes, the presence of PLA_2_ activity was previously reported from the venoms of both the scolopendrid subfamilies Otostigminae (*Otostigmuspradoi*) and Scolopendrinae (*Scolopendra viridicornis* and *S. viridis*) [16,63]. Nevertheless, a PLA_2_, namely Scol/pla, has been characterized from the venom of centipede *S. viridis* [16]. PLA_2_s have also been reported from *S. subspinipes dehaani* [11]. In addition, recently PLA_2_ homologs have been found in the transcriptomes of *S. alternans* and *S. morsitans* as well as in the venom of *S. morsitans* and *E. rubripes* [15].

#### 2.3.5. γ-Glutamyl Transpeptidases

γ-Glutamyl transpeptidases (GGTs) are a well-known family of enzymes, which are reported to be involved in regulation of oxidative stress and xenobiotic detoxification [15,74]. GGT was previously reported from the venoms of parasitoid wasps, which is thought to induce apoptosis of host ovaries by oxidative stress [75,76]. Recently, GGT has been reported to be present in the venom of *S. subspinipes dehaani*, and functionally it is found to induce aggregation of platelets from human and hemolysis of red blood cells from mice and rabbits [11]. According to a previous report [15], transcripts encoding GGT were found in all tested species, albeit only as a single partial contig in both *E. rubripes* and *T. longicornis*. Contrastingly, GGT was highly expressed in the venom glands of *S. alternans*, *S. morsitans*, and *C. westwoodi*. It was revealed that GGT was abundant in the venom of *S. morsitans*. Based on all mass spectrometry experiments, GGT was among the most frequently identified venom proteins from *S. morsitans* and *C. westwoodi*. Thus, GGT seems to be an important component of scolopendrine venoms which was probably recruited into the venom subsequent to the split between two scolopendrid subfamilies approximately 230 Mya [15,19,77].

#### 2.3.6. Other Enzymes

Other enzymes from centipedes have been described in the reports [15,19] some. An enzyme, glucose dehydrogenase, was found to be present in relatively high levels in the venom of all scolopendrids that have thus far been studied. Partial transcripts were also found in *T. longicornis* and *S. alternans*. Type B carboxyl esterase was previously found in the venom of *C. westwoodi* and homologous transcripts identified in the remaining species except *E. rubripes*. Furthermore, a number of isoforms of porphyromonas-type peptidyl arginine deiminase (PPAD) was found in the venom of *T. longicornis* [15].

### 2.4. Enzyme Inhibitors

Recent reports described the presence of potential protease inhibitors in centipede [15,19]. Two cystatin isoforms, containing the characteristic peptidase-interacting sequence Gln-Xaa-Val-Xaa-Gly, as well as the cystatin type-1 like Pro-Gly pair, have been reported to exist in the venom of *E. rubripes*. It was suggested that these venom proteins have retained their ancestral function as peptidase [15]. Trypsin inhibitor has been reported in the venom gland transcriptome of *S. subspinipes dehaani* [11]. Recently, a poor FXa inhibitor, a natural peptide with the sequence of Thr-Asn-Gly-Tyr-Thr (TNGYT), was identified and characterized from the venom of *S. subspinipes mutilans*. This peptide inhibited the activity of FXa in a dose-dependent manner with an IC_50_ value of 74.2 mM [13], indicating that centipede venom may contain protease inhibitors to protect some other important components from degradation.

### 2.5. Anticoagulants or Antithrombotic Peptides

A 25 kDa protein, scolonase, isolated from *S. subspinipes mutilans*, was described mainly as a serine protease. In addition, it was also reported to demonstrate fibrinolytic activity. Experimentally, scolonase was found to convert human Glu-plasminogen to activated plasmin [62]. According to a previous report, centipede acidic protein (CAP) was found to significantly suppress the development of atherosclerosis and improve the hemorheological disturbances as well as histopathological changes in the atherogenic-diet rat model [78]. By transcriptomic analysis, transcripts possessing platelet aggregation, hemolytic as well as anticoagulation activities, have been reported to be present in the venom gland transcriptome of centipede *S. subspinipes dehaani* [11]. Recently, a novel antithrombotic peptide, with a molecular weight of 346 Da, was isolated from *S. subspinipes mutilans*. This short peptide, with the sequence Ser-Gln-Leu (SQL), was found to potently prolong the activated partial thromboplastin time (aPTT) and inhibit platelet aggregation [17].

### 2.6. Centipede Extracts and Anticancer Activity

A few studies indicate the anticancer potential of centipede extracts. Firstly, a study previously reported the presence of an antitumor effect of centipede extract on cervical tumor in mice [79]. Later on, the potential antitumor activity of the alcohol extracts of centipede *S. subspinipes mutilans* (AECS) was investigated, and the results revealed that AECS inhibits A375 cell proliferation in a dose- and time-dependent manner. Moreover, it revealed that the underlying mechanism of AECS-inhibiting A375 cell proliferation was associated with the induction of cell cycle arrest and apoptosis [80]. A recent study reported the anticancer effects of AECS against epidermal growth factor receptor (EGFR)-dependent cancers [81]. AECS was demonstrated to decrease EGFR phosphorylation leading to the down-regulation of the mitogen-activated protein kinase (MAPK) pathway and the phosphatidylinositol 3-kinase/protein kinase B (PI3K/PKB) pathway activity. In addition, AECS was found to induce apoptosis of high-EGFR-expressing cells by decreasing Bcl-2 and increasing BimEL, Bax and Bad expressions. All of them were reported to contribute to the inhibition by AECS on high-EGFR expression cell growth, indicating the efficacy of AECS as a potential strategy against high-EGFR-expressing cancers [81]. Scolopendrasin VII was described as a cationic antimicrobial peptide from centipede *S. subspinipes mutilans*, and further reported to have anticancer activities against U937 and Jurkat leukemia cell lines [53]. MTS assays in the study revealed that scolopendrasin VII decreased the viability of the leukemia cells. Additionally, scolopendrasin VII was found to induce necrosis in the leukemia cells in flow cytometric analysis and acridine orange/ethidium bromide staining [53].

### 2.7. Other Proteins and Substances

In addition to the above, some other components are thought to be present in the venoms of centipedes, although they are not functionally described. According to the previous reports, transferrin was identified in the venoms of centipedes *Ethmostigmus rubripes* *and* *S. morsitans*, and transcripts were found in three other species [15]. Besides centipede β-Pore-forming toxins, LDLA domain-containing proteins as well as some protein families with unknown function have been reported to exist in the transcriptome of centipedes [15]. Not only proteins/peptides, but also functional polysaccharides and several non-peptidic components were also detected and reported in centipede venoms. For instance, lipoproteins, phospholipids, cholesterol, free fatty acids, triglycerides, cholesterol esters, and squalene were found in the venom of *S. morsitans* and reported [61]. 5-Hydroxytryptamine (5-HT or serotonin) and histamine were reported as algesics from centipede venoms, which cause instant, sharp pain [1,82]. Serotonin was also reported to be present in the milked venom of *S. viridicornis* [83]. Additionally, histamine was detected in the venom system of Scolopendridae, including maxilliped extracts of *S. subspinipes* [84]. Disintegrin activity was also found in milked venom of *S. subspinipes* collected in Manipur, India [85]. Recently, a raw polysaccharide and three groups of polysaccharides were isolated from the Chinese red-headed centipede *S. subspinipes mutilans*. Group 1 with the molecular weight of 33.1 kDa was reported to showan inhibitory effect on tumor cells. In the MTT assay performed in the presence of polysaccharide (3.13 µg/mL), group 1 was found to show an effect on “HeLa” cells with an inhibition rate of 60.8% [86].

## 3. Therapeutic Potential of Centipede Venoms and Their Components

The vast majority of centipede toxins remain functionally uncharacterized, and accordingly little is known about the venom components of centipedes. However, venoms as well as their components that have been identified, especially most of the functionally described components from centipedes (Table 1), could be exploited for drug development. In addition, the development of increasingly sensitive and accurate analytical tools can be used to perform more extensive research on centipede venoms, which would enable scientists to find many more pharmacologically important components from centipedes. Here, we attempt to describe the possible therapeutic application of centipede venoms and their pharmacologically important components.

**Table 1:** Functionally described components from centipedes.

Described Components	Centipede Species	Activities	References
RhTx	Ssm	Activator of TRPV1 channel	[29]
µ-SLPTX-Ssm1a	Ssm	Inhibitor of (TTX-S) Na^+^ channel	[12]
κ-SLPTX-Ssm1a	Ssm	Inhibitor of K^+^channel	[12]
κ-SLPTX-Ssm2a	Ssm	Inhibitor of K^+^channel	[12]
κ-SLPTX-Ssm3a	Ssm	Inhibitor of K^+^channel	[12]
ω-SLPTX-Ssm1a	Ssm	Activator of Ca^2+^ channel	[12]
ω-SLPTX-Ssm2a	Ssm	Inhibitor of Ca^2+^ channel	[12]
μ-SLPTX-Ssm6a	Ssm	Potent inhibitor of Na_V_1.7	[9]
SsmTx-I	Ssm	A selective blocker of K_V_2.1 channel	[6]
GenBank accession No.: KC144287, KC144104, KC144040, KC144849, KC144556, KC144606, KC144226	Ssd	Inhibitor of K^+^ channel	[11]
GenBank accession No.: KC144347, KC144448, KC144967, KC145039	Ssd	Inhibitor of Ca^2+^ channel	[11]
GenBank accession No.: KC144793	Ssd	Inhibitor of Na^+^ channel	[11]
Scolopin 1	Ssm	AMP Moderate hemolytic activity	[47]
Scolopin 2	Ssm	AMP Moderate hemolytic activity	[47]
Lactoferricin B like peptide (LBLP)	Ssm	AMP	[49]
Scolopendin 1	Ssm	AMP	[45]
Scolopendin 2	Ssm	AMP	[50]
Scolopendrasin I	Ssm	AMP	[51]
Scolopendrasin II	Ssm	AMP	[52]
Scolopendrasin VII	Ssm	AMP Anticancer activities against U937 and Jurkat leukemia cells	[53]
Ten (10) synthetic peptides (Assigned no name)	Ssm	AMP	[31]
Three (3) naphthylamidase-type aminopeptidases (enzymes)	Ssm	Proteases	[61]
Endo- as well as exo-peptidases (enzymes)	Ssm	Proteases Fibrinolytic activity	[1]
Scolonase (enzyme)	Ssm	Serine protease Fibrinolytic activity Plasmin activator	[62]
Trypsin-like S1 family (enzyme)	Scolopendrid	Proteases	[15]
Subtilisin-like S8 family (enzyme)	Scolopendrids	Proteases	[15]
Enzymes (KC145121,KC145122)	Ssd	Trypsin-like proteases	[11]
~10% of venom proteins	*T. longicornis*	Astacin-like metalloendoproteases	[15]
Enzymes	*S. morsitans*	Non-specific esterases	[1,61,70]
Enzyme	*C. westwoodi*	Type B carboxyl esterase	[15]
A number of isofroms	*T. longicornis*	Porphyromonas-type peptidyl arginine deiminase (PPAD)	[15]
Enzymes	*Ot. pradoi*, *S. viridicornis*, *S. viridis*	PLA_2_	[15,16,63]
Scol/pla	*S. viridis*	PLA_2_	[16]
Transcripts	*S. alternans*, Ssm, *E. rubripes*	PLA_2_ homologs	[15]
Enzyme	Ssd	γ-Glutamyl transpeptidase (GTT) Inducer of platelet aggregation	[15]
Transcripts	Five species tested in reference	γ-Glutamyl transpeptidase (GTT)	[15]
Enzyme	Scolopendrids	Glucose dehydrogenase	[15]
Cystatin type-1	*E. rubripes*	Peptidase inhibitors	[15]
Two cystatin isoforms	*E. rubripes*	Peptidase inhibitors	[15]
A natural peptide	Ssm	FXa inhibitor,	[13]
GenBank accession No.: KC144061	Ssd	Trypsin inhibitor	[11]
Centipede acidic protein (CAP)	Scolopendrids	Suppressor of atherosclerosis Improver of hemorheological disturbances	[78]
Antithrombotic peptide	Ssm	Inhibitor of platelet aggregation	[17]
GenBank accession No.: KC144034	Ssd	Platelet aggregation and Hemolytic activity	[11]
GenBank accession No.: KC144430	Ssd	Anticoagulation	[11]
Centipede extracts	Ssm	Antitumor effect on cervical tumor in mice. Inhibitor of A375 cell proliferation. Anticancer effects against epidermal growth factor receptor (EGFR)-dependent cancers. Inducer of high-EGFR expression cell apoptosis	[79,80,81]
Serotonin	*S.* *viridicornis*	Algesics that cause instant sharp pain	[1,82,83]
Histamine	*S.* *subspinipes*	Algesics that cause instant sharp pain	[1,82,84]
Polysaccharide	Ssm	Inhibitor of tumor cells	[86]
Transferrin	*E. rubripes*, *S. morsitans*	Potential antimicrobial component	[15]

Ssm indicates *Scolopendra subspinipes mutilans*; Ssd indicates *Scolopendra subspinipes dehaani*.

Because the Nav1.7 channels are involved in pain sensation, the novel analgesics, μ-SPTX-Ssm6a, identified from the Chinese red-headed centipede, targeting Na_V_1.7, may be a suitable therapeutic agent for treating a wide range of human pain pathologies [9]. Furthermore, Na_V_ channels have also been reported as the target of several classes of therapeutics such as local anesthetics (e.g., lidocaine and ropivacaine), antiarrhythmics (e.g., mexiletine and flecainide), and anticonvulsants used to treat epilepsy or bipolar disorder (e.g., lamotrigine, carbamazepine, and phenytoin) [28,87,88]. Thus, discovery of Na_V_ channel modulators from centipede venoms could be used as potential therapeutics for pain as well as other related diseases; moreover, it could help to find a new gateway for molecular design and drug development.

Similar to Na_V_, K^+^ channels (K_V_) have shown promise as potential therapeutic targets for the treatment of some human diseases ranging from asthma [89] diabetes, angina, cardiac ischemia and hypertension [90] to chronic inflammation, autoimmune disease and cancer [91]. Therefore, K_V_ channel inhibitors from centipede discovered thus far, such as κ-SLPTX-Ssm1a, κ-SLPTX-Ssm2a, κ-SLPTX-Ssm3a, SsmTx-I, as well as other K^+^ channel inhibitors reported from *S. subspinipes dehaani*, could be screened for suitable therapeutic drugs to treat the diseases mentioned above.

Furthermore, voltage gated calcium channels are reported as an analgesic target. Specifically, the T-type calcium channel is involved in cell proliferation in leukemia cells [92], while Ca_V_2.2, is involved in nociceptive pathways [93]. Thus, peptides showing function on Ca_V_ channels, ω-SLPTX-Ssm1a and ω-SLPTX-Ssm2a may be investigated deeply to find their specific functions on T-type as well as Ca_V_2.2 channels and subsequently screened for identification of a suitable lead molecule to treat nociception or leukemia. Moreover, venom peptides have been reported as a rich source of Ca_V_2.2 channel blockers [93]. It indicates the presence of Ca_V_2.2 blockers in the venom of centipedes, which could be identified, purified, characterized and screened for therapeutic use. Most importantly, RhTx, as an agonist, activates the TRPV1 channel through heat-activation machinery, which could be used as a model to study the role of TRPV1 in pain sensation as well as development of new pain killers. Like other venomous animals, centipede venom may also contain different types of ion channel modulators which remain as of yet unidentified, but could be identified and used for related therapeutic purposes according to their functions.

In recent years, microbial resistance to antibiotics has increased, thereby resulting in the invalidation of antibiotic drugs used clinically [50,94], subsequently leading to a failure of the drugs to interact with their target [95,96]. In this regard, discovery of novel antimicrobial compounds show great promise. Like other venomous animals, antimicrobial peptides have been identified from the venoms of centipede. Therefore, the antimicrobial peptides from centipedes reported to date could be usefully exploited to develop antibiotic drugs, which could replace the drugs that no longer defend against infectious microorganisms.

Proteases, protease inhibitors, and protease-activated receptors have been intensively investigated in the peripheral brain for their roles in a wide range of processes, such as coagulation, inflammation, and digestion [97,98,99,100,101]. However, interest in characterizing new protease inhibitors (PIs) and understanding their physiological significance has increased due to their biological relevance for living processes, such as blood coagulation system, complement cascade, apoptosis, cell cycle and hormone-processing pathways [102,103,104,105]. Furthermore, dysregulations or alterations in the regulation of these enzymes can lead to several pathological conditions, such as cancer, arthritis, osteoporosis, neurodegenerative and cardiovascular diseases [106,107]. Importantly, protease inhibitors, trypsin inhibitors, such as ulinastatin and aprotinin, are already being clinically used in anti-inflammatory therapy [108]. Likewise, the protease inhibitors identified from centipedes might have therapeutic potential in anti-inflammatory therapy or other serine protease related diseases.

The reports as described above indicated that alcohol extracts of the centipede *S. subspinipes mutilans* (AECS) inhibits A375 cell proliferation, as well as shows anticancer activity against epidermal growth factor receptor (EGFR)-dependent cancers. Specifically, scolopendrasin VII has the anticancer activities against U937 and Jurkat leukemia cell lines. These studies suggest that centipede contains anticancer peptides which may be purified and used as potential therapeutic agents for administration in human cancers, and may help develop new anticancer agents.

According to previous reports in invertebrates, proteins belonging to the transferrin superfamily have been implicated in pathways involved in the reaction to secondary infections by binding iron and creating an environment low in free iron that inhibits bacterial survival [15,109]. Therefore, it could be stated that centipede venom transferrin potentially function as an antimicrobial agent in the venom gland [15], which might be purified and used in antimicrobial drug discovery.

## 4. Conclusions

Centipede venoms constitute a rich source of pharmacologically important proteins and peptides. The components identified and described thus far could be useful for therapeutic applications. However, all the studies indicate that there are many other components left unidentified and uncharacterized. Centipede venom must therefore be studied more extensively for the discovery of additional therapeutic components. As of yet, only a handful of venom-derived drugs have been approved for use in treatment for pathophysiological conditions, including chronic pain, diabetes and hypertension. A few dozen other venom-derived peptides and proteins with excellent pharmacological activities, however, are presently undergoing clinical trials. Therefore, we believe that centipede venom could be an innovative source of peptides and proteins with excellent properties for the development of effective drugs.
